# Vitamin D deficiency is associated with high prevalence of diabetes in Kuwaiti adults: results from a national survey

**DOI:** 10.1186/s12889-016-2758-x

**Published:** 2016-02-01

**Authors:** Fang Fang Zhang, Suad Al Hooti, Sameer Al Zenki, Husam Alomirah, Kazi M Jamil, Aravinda Rao, Nasser Al Jahmah, Edward Saltzman, Lynne M. Ausman

**Affiliations:** 1 Friedman School of Nutrition Science and Policy, Tufts University, 150 Harrison Ave, Boston, MA 02111 USA; 2 Kuwait Institute for Scientific Research, PO Box 24885, Safat, 13109 Kuwait; 3 Ministry of Health, Medical Laboratories Services, Sabah Hospital Laboratories, PO Box 24225, Safat, 13103 Kuwait

**Keywords:** Vitamin D deficiency, 25(OH)D, Diabetes, Prediabetes

## Abstract

**Background:**

Vitamin D homeostasis may play a critical role in glucose metabolism. Little is known on vitamin D deficiency and its association with diabetes in countries of the Arabia Gulf where the population is experiencing a rapid increase in the incidence of diabetes.

**Methods:**

In a cross-sectional study of 960 adults enrolled in the first National Nutrition Survey of the State of Kuwait (NNSSK), we examined vitamin D status in association with the prevalence of diabetes and prediabetes. Vitamin D status was measured by serum levels of 25-hydroxyvitami D (25(OH)D). Prevalences of diabetes and prediabetes were determined based on fasting glucose and HbA1C levels.

**Results:**

The median level of serum 25(OH)D in Kuwaiti adults was 13.8 ng/ml. Approximately 56 % of the Kuwaiti adults had vitamin D inadequacy (25(OH)D = 12–19.9 ng/ml), and 27 % had vitamin D deficiency (25(OH)D < 12 ng/ml). The prevalences of prediabetes and diabetes were 40 and 27 %, respectively. Vitamin D inadequacy (OR = 1.7, 95 % CI: 1.0–2.9) and deficiency (OR =2.0, 95 % CI: 1.1–3.3) was each associated with about two-fold increased odds of prediabetes compared to sufficient vitamin D status (25(OH)D ≥20 ng/ml). Vitamin D inadequacy (OR =2.1, 95 % CI: 1.2–3.7) and deficiency (OR =2.0, 95 % CI: 1.1–3.9) were also associated with two-fold increased odds of diabetes.

**Conclusions:**

Data from Kuwaiti’s first nutrition nutritional survey suggests a very high prevalence of vitamin D deficiency in Kuwaiti adults. Associations of low vitamin D status and high prevalence of diabetes point to the need of continuous monitoring of vitamin D status and further evaluating potential health consequences in this high-risk population.

**Electronic supplementary material:**

The online version of this article (doi:10.1186/s12889-016-2758-x) contains supplementary material, which is available to authorized users.

## Background

The incidence of diabetes and other metabolic disorders is increasing at an alarming rate in countries of the Arabian Gulf, possibly due to dramatic changes in lifestyle and food intake patterns since the discovery of oil in the 1950s [1, 2]. The National Nutrition Surveillance System data from 1998 to 2009 revealed that the prevalence of obesity reached 77 % in Kuwaiti adults [3]. The prevalence of diabetes and metabolic syndrome is also higher in the State of Kuwait than in many developed countries, placing “a wake-up call for public health intervention” [2, 4].

A growing literature suggests that vitamin D homeostasis may play a role in the etiology of type 1 and type 2 diabetes [5–7]. Animal and cell studies discovered that vitamin D affects insulin secretion through binding of its active form to vitamin D receptors in pancreatic beta cells [8–11]. Vitamin D also affects insulin resistance by stimulating the expression of insulin receptors [12], or indirectly by regulating calcium homeostasis [13]. Despite proposed biological mechanisms, observational studies and clinical trials in humans did not provide consistent evidence regarding the association between vitamin D status and diabetes [7, 14, 15]. Furthermore, most studies were conducted in populations of North America and few studies investigated vitamin D deficiency in association with diabetes in other racial/ethnic groups for whom the vitamin D status could be substantially lower than that in non-Hispanic whites [16].

In Middle East, despite ample sunshine, vitamin D deficiency is common due to cultural practice and dark skin color [17]. Few studies from Middle East have examined the association between vitamin D status and diabetes [18–20]. In particular, evidence is lacking from a nationally representative sample of Middle East population. Here we report findings from the first national survey in Kuwaiti adults of vitamin D status and its association with the prevalence of diabetes.

## Methods

### Study participants

Kuwait Institute for Scientific Research in association with the Ministry of Health in Kuwait conducted a National Nutrition Survey of the State of Kuwait (NNSSK) between July 2008 and November 2009 to assess the nutrition status and the prevalence of major nutrition-related chronic diseases in the Kuwaiti population [2, 4]. The NNSSK is a household based cluster survey. Specifically, a stratified probability cluster sampling method was used to obtain a representative national sample of Kuwaiti households from 82 clusters distributed among the six governorates of Kuwait in proportion to population size. Initially, 1,693 households and 7,547 selected household members gave consent to participate in the survey. Of those who consented, 545 households with a total of 1,830 household members (48 % males and 52 % females) responded to the questionnaire based interviews conducted at seven primary health care clinics of the Ministry of Health located at various districts of Kuwait. Structured questionnaires were administered to survey subjects to collect data on socioeconomic status, medical history and risk behaviors such as smoking and physical activity. Dietary intake was assessed by a single 24-h diet recall. Subjects were measured for weight, height, and blood pressures. Fasting blood samples were collected to assess biomarkers for nutrition intake and chronic diseases. Among all survey participants, 1,021 were adults age ≥20 years old. For the current analysis, 960 (94.0 %) adults who had blood measurement of vitamin D were included as study participants.

The study has been approved by the institutional review boards of Tufts University and Kuwait Institute for Scientific Research.

### Vitamin D status

Serum concentration of 25-hydroxyvitamin D (25(OH)D) was assessed for vitamin D status. Fasting blood samples were collected by qualified phlebotomists and transported to the Sabah Hospital National Reference Laboratory for processing, storage and analysis. Serum 25(OH)D concentration was analyzed on a Cobas e601 immunoassay analyzer (Roche Diagnostics, USA) using manufacturer’s kit. The kit is based on the electrochemiluminescence principle, using streptavidin coated paramagnetic microparticles as universal solid phase reagent, and biotin bound ligand and Ruthenium Bipyridyl (Ru(bpy)_3_^2+^ complex as the label. The reagent kits are pre-calibrated and the master curve is readjusted before each batch of assay using a 2-point calibrator set provided in the kit. Manufacturer’s control sera (Preci-Control, Roche Diagnostics) were used as internal control and Bio-Rad EQAS and Randox International Quality Assurance Scheme were used for external quality assurance. The serum sample was incubated with Biotin –vitamin D and polyclonal anti-vitamin D_3_ antibody-(Ru(bpy)_3_^2+^ complex. Solid phase is added and after further incubation, the bound complex is captured and washed and signal generated. The assay was standardized against LC-MS/MS method. The inter-assay CV was 5–8 % and the assay range was 4–100 ng/ml.

### Prevalence of diabetes and prediabetes

Diabetes and prediabetes were defined based on fasting glucose and hemoglobin A1c (HbA1C) levels and self-reported diagnosis of diabetes or use of medications to control diabetes, according to the current diagnosis criteria published by the American Diabetes Association (ADA) in 2012 [21]. Specifically, a subject was defined as having diabetes if fasting glucose ≥7.0 mmol/L or HbA1C ≥ 6.5 %, or if a subject reported a physician’s diagnosis of diabetes or use of medications to control diabetes. A subject was defined as having prediabetes if fasting glucose = 5.6–6.9 mmol/L or HbA1C = 5.7–6.4 %. Fasting glucose was analyzed on Dade-Behrin (Siemens) Dimension RxL automated clinical chemistry analyzer, using the manufacturer’s kit. Bi-level internal quality control materials were run in the beginning, middle and end of the batch. Bio-Rad external Quality Assurance scheme provided external quality assurance. Hexokinase-glucose–6-phosphate dehydrogenase method was used by the kit. The inter-assay CV was 1.5–3.5 % and the assay range was 0–27.8 mmol/L. Hemoglobin A1C was measured on Roche Cobas Integra 400. The blood was hemolysed automatically at low osmotic pressure. Calibration was done using a synthetic N-terminal Hemogolobin A_1c_ polypeptide. The non-linear standard curve was transformed by using logit-log plot. The inter-assay CV was 2.5 %, and the assay range was 1.4-47umol/L (24–758 ml/L).

### Statistical analysis

We first evaluated characteristics of the study participants by vitamin D status and by diabetes status. Vitamin D status was categorized into deficiency (<12 ng/ml), inadequacy (12–19.9 ng/ml) or sufficiency (≥20 ng/ml) based on serum concentration of 25(OH)D according to the cut off values defined in the Dietary Reference Intakes released by the Institute of Medicine in the United States [22, 23]. Differences in continuous variables were tested using Analysis of Variance (ANOVA), and differences in categorical variables were tested using a Chi-square test. Participants were considered to be physically active if they self-reported moderate physical activity ≥150 min/week or strenuous physical activity ≥75 min/week and to be sedentary otherwise [24]. Body mass index (BMI) was calculated based on weight and height using the standard formula, i.e., BMI = weight (kg)/height (m)^2^. Weight status was categorized as underweight if BMI < 18.5 kg/m^2^, normal weight if BMI =18.5–24.9 kg/m^2^, overweight if BMI = 25–29.9 kg/ m^2^, and obese if BMI ≥ 30 kg/m^2^. Because only 1.1 % of the Kuwaiti adults were underweight, they were combined into a single group with those with normal weight. For dietary intake of vitamin D and calcium, we excluded 7 participants who reported extreme total energy intake. Extreme energy intake was defined as the log-transformed total energy intake > mean + 3 × standard deviation or < mean – 3 × standard deviation. After adjusting for total energy intake, dietary intake of vitamin D and calcium was categorized into low and high intake group based on the median. Because dose was not captured for vitamin supplements, supplemental use of vitamin D and calcium was categorized into users and nonusers. Season was categorized into spring (Ferburary 16–May 20), summer (May 21–Novemer 4), fall (November 5–December 5) and winter (December 6–Ferburary 15). Fall and winter seasons were combined into one group because of similar vitamin D concentrations and short duration of fall season.

The association between vitamin D status and diabetes was examined using multinomial logistic regression models. We first compared the prevalence odds of prediabetes and diabetes among participants with different vitamin D status. Participants with sufficient vitamin D status (≥20 ng/ml) were treated as the reference group. Odds ratios (PORs) and 95 % confidence intervals (CIs) were first adjusted for age (Model I) and then additionally adjusted for a prior set of confounders including sex, body mass index, physical activity, smoking status, dietary intake and supplemental use of vitamin D and calcium, and season of blood draw (Model II). We also compared the prevalence odds of vitamin D inadequacy and deficiency among nondiabetic, prediabetic, and diabetic participants after adjusting for age and other confounders. In addition, we assessed whether the associations between vitamin D status and diabetes were modified by weight status and dietary intake and supplemental use of vitamin D and calcium. The significance of multiplicative interaction was examined using the likelihood ratio test.

Sample weighting techniques and post-stratification adjustments were made to match it to the 2005 Kuwait census population and to reduce non-response rate or selection biases. A raking method was used to calculate non-response-adjusted weights producing a final set of person weights to perform data analyses [25, 26]. Sampling weights were adjusted in all analyses to account for the complex sampling design of the survey. All statistical analyses were performed using SAS (version 9.3; SAS Institute, Cary, NC).

## Results

Of 960 Kuwaiti adults who had serum concentration of vitamin D, the mean age was 43.6 years (SD = 14.6), and 45.4 % were male. The mean body mass index (BMI) was 29.9 kg/m^2^ (SD = 6.4), with 33.6 % being overweight and 45.0 % being obese. Self-reported physical activity indicated that a high proportion of Kuwaiti adults was sedentary, with 84.1 % not meeting the 2008 Physical Activity Guidelines for Americans (i.e. ≥150 min/week moderate physical activity or ≥75 min/week vigorous physical activity) [27]. About one-third of the study participants were former (12.7 %) or current (19.6 %) smokers. The median dietary intakes of vitamin D and calcium from food sources were 20.9 IU/day and 342.2 mg/day respectively. The use of vitamin supplement was uncommon in Kuwaiti adults: only 4.5 % of the participants reported the use of vitamin D supplement and 9.3 % reported the use of calcium supplement.

The median level of serum 25(OH)D was 13.8 ng/ml (IQR = 7.7). Over one-third of the Kuwaiti adults (36.3 %) had vitamin D deficiency (25(OH)D <12 ng/ml), and nearly half (46.6 %) had vitamin D inadequacy (25(OH)D =10–19.9 ng/ml). Participants who were vitamin D deficient or inadequate, compared to participants who had sufficient level of vitamin D (25(OH)D ≥ 20 ng/ml), were significantly younger (mean age = 39.3 and 44.2 vs. 49.6 years, *p* < 0.0001), and had a higher BMI (mean BMI =30.4 and 30.1 vs. 28.3 kg/m^2^, *p* = 0.001) and a lower dietary intake of vitamin D (median intake = 14.8 and 19.9 vs. 26.7 IU/day/1,000 kcal, *p* = 0.02) and calcium (median = 296.8 and 319.2 vs. 368.8 mg/day/1,000 kcal, *p* = 0.004) (Table 1). They were also more likely to be females (67.2 and 46.8 vs. 49.1 %, *p* < 0.0001) and nonsmokers (75.3 and 64.0 vs. 61.8 %, *p* = 0.005), and receive a higher level of education (57.5 and 49.7 vs. 42.3 %, p < 0.0001), and were less likely to be users of vitamin D (2.0 and 4.3 vs. 10.3 %, *p* = 0.001) or calcium supplements (5.5 and 9.2 vs. 17.6 %, *p* < 0.0001), and to have blood drawn during summer or fall season (80.5 and 85.4 vs. 94.5 %, *p* = 0.0002). No significant difference in the level of physical activity was found by vitamin D status.

**Table 1:** Characteristics of Kuwaiti Adults by Vitamin D Status, NNSSK 2008–2009

	Vitamin D status (Serum 25(OH)D)			
	Sufficient (≥20 ng/ml) (*N* = 165)	Inadequate (12–19.9 ng/ml) (*N* = 447)	Deficient (<12 ng/ml) (*N* = 348)	*P* value
Age *(year),* mean (SD)	49.6 (15.2)	44.2 (14.5)	39.3 (12.7)	<0.0001
Sex, *N* (%)				
Male	84 (50.9)	238 (53.2)	114 (32.8)	<0.0001
Female	81 (49.1)	209 (46.8)	234 (67.2)	
Education, *N* (%)				
Less than high school	70 (42.4)	146 (32.7)	76 (21.8)	<0.0001
High school	17 (10.3)	79 (17.7)	72 (20.7)	
College or higher	78 (42.3)	222 (49.7)	200 (57.5)	
BMI (*kg/m* ^*2*^ *),* mean (SD)	28.3 (5.1)	30.1 (6.5)	30.4 (6.8)	0.001
*N* (%)				
<25	44 (26.7)	91 (20.5)	70 (20.2)	0.01
25–29	67 (40.6)	149 (33.6)	105 (30.3)	
≥30	54 (32.7)	204 (46.0)	172 (49.6)	
Physical activity^a^, *N* (%)				
Sedentary	140 (84.9)	367 (82.5)	297 (85.8)	0.42
Active	25 (15.2)	78 (17.5)	49 (14.2)	
Smoking status, *N* (%)				
Nonsmokers	102 (61.8)	286 (64.0)	262 (75.3)	0.005
Former smokers	24 (14.6)	63 (14.1)	35 (10.1)	
Current smokers	39 (23.6)	98 (21.9)	51 (14.7)	
Season of blood draw^b^, *N* (%)				
Winter/Spring	9 (5.5)	64 (14.6)	66 (19.5)	0.0002
Summer/fall	155 (94.5)	374 (85.4)	273 (80.5)	
Dietary intake of vitamin D (*IU/day per 1,000 kcal)*				
Median (IQR)	26.7 (57.3)	19.9 (47.1)	14.8 (41.0)	0.02
*N* (%)				
Low (<20.9)	195 (51.5)	171 (53.9)	137 (57.3)	0.02
High (≥20.9)	184 (48.5)	146 (46.1)	102 (42.7)	
Supplemental use of vitamin D, *N* (%)				
No	148 (89.7)	428 (95.8)	341 (98.0)	0.0001
Yes	17 (10.3)	19 (4.3)	7 (2.0)	
Dietary intake of calcium (*mg/day per 1,000 kcal)*				
Median (IQR)	368.8 (265.4)	319.2 (236.8)	296.8 (208.3)	0.0004
*N*(%)				
Low (<348.2)	217 (55.9)	154 (47.5)	108 (44.4)	0.01
High (≥348.2)	171 (44.1)	170 (52.5)	135 (55.6)	
Supplemental use of calcium, *N* (%)				
No	136 (82.4)	406 (90.8)	329 (94.5)	<0.0001
Yes	29 (17.6)	41 (9.2)	19 (5.5)	

^a^A subject was defined as being physically active if having moderate physical activity ≥150 min/week or strenuous physical activity ≥75 min/week, and physically inactive otherwise, according to the 2008 CDC physical activity guidelines [1]

^b^Spring and summer seasons were Feb.16-Nov.4, and fall and winter seasons were Nov.5–Feb.15

About one-fourth (25.5 %) of the Kuwaiti adults had diabetes and one-third (33.9 %) had prediabetes. Diabetic and prediabetic participants, compared to nondiabetic participants, were significantly older (54.7 and 45.5 vs. 34.6 years, *p* < 0.0001), had a higher BMI (31.5 and 31.1 vs. 27.9 kg/m^2^, *p* < 0.0001), and tended to have high dietary intake of vitamin D (57.3 and 46.1 vs. 48.5 %, *p* = 0.02) and calcium (55.6 and 52.5 vs. 44.1 %, *p* = 0.01). Diabetic and prediabetic participants also had a lower percentage of completing college education (35.3 and 54.9 vs. 60.3 %, *p* < 0.0001) and were less likely to be physically activity compared to nondiabetic participants (9.0 and 12.7 vs. 23.1 %, *p* < 0.0001) (Table 2).

**Table 2:** Characteristics of Kuwaiti Adults by Diabetes Status, NNSSK 2008-2009

	Nondiabetic (*N* = 388)	Prediabetic^a^ (*N* = 324)	Diabetic^a^ (*N* = 244)	*P* value
Age (*yr),*mean (SD)	34.6 (11.5)	45.5 (13.0)	54.7 (11.4)	<0.0001
Sex, *N* (%)				
Male	165 (42.5)	148 (46.0)	122 (50.0)	
Female	223 (57.5)	175 (54.0)	122 (50.0)	0.18
Education, *N* (%)				
Less than high school	78 (20.1)	91 (28.1)	122 (50.0)	
High school	76 (19.6)	55 (17.0)	36 (14.7)	
College or higher	234 (60.3)	178 (54.9)	86 (35.3)	<0.0001
BMI (kg/m2), mean (SD)	27.9 (6.3)	31.1 (6.5)	31.5 (5.7)	<0.0001
*N* (%)				
<25	130 (33.7)	48 (14.9)	27 (11.1)	
25–29	145 (37.6)	102 (31.6)	73 (30.0)	
≥30	111 (28.8)	173 (53.5)	143 (58.9)	<0.0001
Physical activity^b^, *N* (%)				
Sedentary	296 (76.9)	282 (87.3)	222 (91.0)	
Active	89 (23.1)	41 (12.7)	22 (9.0)	<0.0001
Smoking status, *N* (%)				
Nonsmokers	264 (68.0)	218 (67.3)	164 (67.2)	
Former smokers	35 (9.0)	44 (13.6)	43 (17.6)	
Current smokers	89 (22.9)	62 (19.1)	37 (15.2)	0.01
Season of blood draw^c^, *N* (%)				
Winter/Spring	201 (51.8)	192 (59.3)	135 (55.6)	
Summer/fall	187 (48.2)	132 (40.7)	108 (44.4)	0.14
Dietary intake of vitamin D, *IU/day per 1,000 kcal*				
Median (IQR)	17.2 (43.7)	16.4 (41.8)	26.2 (57.1)	0.04
*N* (%)				
Low (<20.9)	195 (51.5)	171 (53.9)	102 (42.7)	
High (≥20.9)	184 (48.5)	146 (46.1)	137 (57.3)	0.02
Supplemental use of vitamin D, *N* (%)				
No	375 (96.7)	305 (94.1)	233 (95.5)	
Yes	13 (3.4)	19 (5.9)	11 (4.5)	0.27
Dietary intake of calcium, *mg/day per 1,000 kcal*				
Median (IQR)	303.1 (216.3)	328.5 (230.3)	357.7 (259.8)	0.003
*N* (%)				
Low (<348.2)	217 (55.9)	154 (47.5)	108 (44.4)	
High (≥348.2)	171 (44.1)	170 (52.5)	135 (55.6)	0.01
Supplemental use of calcium, *N* (%)				
No	356 (91.8)	294 (90.7)	218 (89.3)	
Yes	32 (8.3)	30 (9.3)	26 (10.7)	0.59

^a^According to the American Diabetes Association’s criteria for the diagnosis of diabetes [2], a subject was defined as having diabetes if fasting glucose ≥7.0 mmol/L or HbA1c ≥ 6.5 %, or if he/she reported a physician diagnosis of diabetes or use of medications to control diabetes. A subject was defined as having prediabetes if fasting glucose = 5.6–6.9 mmol/L or HbA1c = 5.7–6.4 %

^b^A subject was defined as being physically active if having moderate physical activity ≥150 min/week or strenuous physical activity ≥75 min/week, and physically inactive otherwise, according to the 2008 CDC physical activity guidelines [1]

^c^Spring and summer seasons were Feb.16–Nov.4, and fall and winter seasons were Nov.5-Feb.15

After multivariate adjustment, serum concentration of vitamin D was significantly lower in prediabetic and diabetic participants (39.6 and 39.8 ng/ml) than nondiabetic participants (42.9 ng/ml, *p* = 0.01). When the prevalences odds of prediabetes and diabetes were evaluated by vitamin D status in multinomial logistic regression models adjusting for age, vitamin D inadequacy was associated with 70 % increased odds of prediabetes (OR = 1.9, 95 % CI: 1.2–3.0), and vitamin D deficiency was associated with two-fold increased odds of prediabetes (OR = 2.1, 95 % CI: 1.3–3.5), and these associations were dose-dependent (P _trend_ = 0.008). Additional adjustment for education, body mass index, smoking status, physical activity, dietary and supplemental use of vitamin D and calcium, and season of blood draw yielded similar results: vitamin D inadequacy was associated with 70 % increased odds of prediabetes (OR = 1.7, 95 % CI: 1.0–2.9), and vitamin D deficiency was associated with two-fold increased odds of prediabetes (OR = 2.0, 95 % CI: 1.1–3.3, P _trend_ = 0.03). Vitamin D inadequacy and vitamin D deficiency were also associated with two-fold increased odds of diabetes (OR = 2.1, 95 % CI: 1.2–3.7 and OR = 2.0, 95 % CI: 1.1–3.9, respectively, P _trend_ =0.06) (Table 3). Similarly, prediabetes and diabetic participants were more likely to have vitamin D inadequacy or deficiency compared to nondiabetic participants (Additional file 1: Table S1).

**Table 3:** Association between Serum 25(OH)D and Prevalence of Diabetes and Prediabetes in Kuwaiti Adults, NNSSK 2008-2009^a^

	Nondiabetic	Prediabetic	OR (95 % CI)		Diabetic	OR (95 % CI)^b^	
	*N* (%)	*N* (%)	Model I^b^	Model II^c^	*N* (%)	Model I^b^	Model II^c^
Vitamin D Status							
Sufficiency (25(OH)D ≥ 20 ng/ml)	66 (40.2)	50 (30.5)	1.0	1.0	48 (29.3)	1.0	1.0
Inadequacy (25(OH)D =12–19.9 ng/ml)	167 (37.4)	149 (33.3)	1.9 (1.2–3.0)	1.7 (1.0–2.9)	131 (29.3)	2.4 (2.4–4.2)	2.1 (1.2–3.7)
Deficiency (25 (OH)D <12 ng/ml)	155 (44.9)	125 (36.2)	2.1 (1.3–3.5)	2.0 (1.1–3.3)	65 (18.8)	2.1 (1.2–3.8)	2.0 (1.1–3.9)
			P _trend_ = 0.008	P _trend_ = 0.03		P _trend_ = 0.046	P _trend_ = 0.06

^a^According to the American Diabetes Association’s criteria for the diagnosis of diabetes [2], a subject was defined as having diabetes if fasting glucose ≥7.0 mmol/L or HbA1c ≥ 6.5 %, and having prediabetes if fasting glucose = 5.6–6.9 mmol/L or HbA1c = 5.7–6.4 %

^b^Model I: Odds ratios (ORs) and 95 % confidence intervals (CIs) were adjusted for age

^c^Model II: Odds ratios (ORs) and 95 % confidence intervals (CIs) were additionally adjusted for sex, education (less than high school, high school, and college or higher), body mass index (continuous), smoking status (current, former, and nonsmokers), physical activity (active *vs*. sedentary), dietary intake of vitamin D and calcium (high *vs*. low according to median), supplemental intake of vitamin D and calcium (yes *vs*. no), and season of blood draw (summer/spring *vs.* winter/fall)

Results from interaction analyses indicated that participants who were deficient/inadequate in vitamin D and overweight/obese had more than three-fold increased odds of diabetes or prediabetes compared to those who had sufficient vitamin D status and were not overweight/obese (OR = 3.6, 95 % CI: 1.8–7.1). Participants who were nonusers of calcium supplement when they were also deficient/inadequate in vitamin D had nearly three-fold increased odds of diabetes or prediabetes compared to those who were sufficient in vitamin D and used calcium supplement (OR = 2.8, 95 % CI:1.3–5.8). However, none of the multiplicative interactions reached statistical significance. Because only a small percentage of the participants used vitamin D supplements, we were not able to evaluate whether the associations differed by supplemental use of vitamin D. Dietary intake of vitamin D and calcium did not modify the associations (Additional file 1: Table S2).

## Discussion

In a population in the Middle East where the prevalence of diabetes has been rising markedly in the past decade, we found vitamin D deficiency is highly prevalent in Kuwaiti adults, and low vitamin D status was associated with a high prevalence of diabetes.

Vitamin D status is often measured by serum levels of 25-hydroxyvitamin D (25(OH)D), the primary circulating form of vitamin D. The Institute of Medicine (IOM) released new Dietary Reference Intakes in 2011 for calcium and vitamin D [22], and defined vitamin D deficiency as serum 25(OH)D <12 ng/ml and vitamin D inadequacy as 25(OH)D =12–19.9 ng/ml. These cut-off values are less strict than the guidelines proposed by the Endocrine Society, for which serum 25(OH)D <30 ng/ml is used to categorize inadequate vitamin D status [28]. Although a low vitamin D status has been reported in populations of Middle East, our study is the first that reported vitamin D status in a national representative sample. Our findings suggested that Kuwaiti adults have an alarmingly low vitamin D status, even based on the less strict IOM definition. Nearly half of the Kuwaiti adults had vitamin D inadequacy (serum 25(OH)D =12–19.9 ng/ml) and more than one-third had vitamin D deficiency (serum 25(OH)D < 12 ng/ml). These together suggest more than 80 % of the Kuwaiti adults are at risk for inadequate vitamin D. This is in contrast to the findings from the United States that about one-quarter of the US population had vitamin D inadequacy and only 8 % were at risk of vitamin D deficiency [29].

The low vitamin D status in Kuwaiti adults may result from reduced exposure to sunshine and low dietary intake of fortified products and dietary supplements. Sun exposure represents the most import source of vitamin D production in human body [30]. Although Kuwait is a country with ample sunshine, the cultural practice of clothing shields most skin from sunshine in particular in women. In this national survey, we observed that vitamin D status was much lower in women than in men. In addition, fortification of dairy products with vitamin D is not mandatory in Kuwait and the consumption of milk is generally very low, possibly due to a high prevalence of lactose intolerance [31]. Although participants who reported the use of vitamin D supplements or had higher dietary intake of vitamin D also had a higher level of serum 25(OH)D, the overall dietary intake of vitamin D was low (median = 21 IU/day per 1,000 kcal) and the use of vitamin D supplement was uncommon (<5 %). Moreover, Kuwait is a country that has been experiencing a marked increase in the prevalence of obesity in the past decade, which can additionally contribute to a low vitamin D status in Kuwaiti adults. These findings point to the need of continuously monitoring vitamin D status in Kuwaiti population and identifying contributing factors.

Interestingly, we observed an advanced age was associated with high vitamin D status in this population. It has to be noted that the adult population in Kuwait are relatively young with a mean age of 43.6 years. Further investigations revealed that older participants were more likely to donate blood in summer/fall seasons when serum 25(OH) was high, and have an overall higher intake of vitamin D from food and supplement than younger participants, which may explain the higher vitamin D status in older adults in Kuwait. Age is also a significant risk factor for diabetes and thus a strong confounder for the association between vitamin D status and prevalence of diabetes. In univariate analysis, a higher prevalence of diabetes was observed in participants with sufficient or inadequate vitamin D status as compared to those deficient in vitamin D. However, adjustment of age revealed the opposite. This is explained by the fact that age served as a negative confounder, i.e. advanced age was negatively associated with vitamin D deficiency but positively with diabetes risk (i.e. a negative confounder) [32]. Therefore, it is essential to adjust the effect of age using multivariate analysis when evaluating vitamin D status and diabetes (potentially other health outcomes) in this population.

Low vitamin D status has been associated with an increased risk of diabetes in populations of North America and Europe [33]. Our results suggest that low vitamin D status is also strongly associated with a high prevalence of diabetes in a Middle East population. The possible mechanisms for this association may include the presence of vitamin D receptors in pancreatic beta cells to which circulating vitamin D binds [34]. Vitamin D has been well recognized for its role in regulating extracellular calcium flux, and insulin secretion is known as a calcium dependent process [35]. Several studies reported an impaired insulin release in association with vitamin D deficiency [11, 36, 37], and vitamin D supplementation has been shown to improve insulin release in randomized controlled trials [38–40]. In addition, vitamin D has been proposed to improve insulin resistance by stimulating the expression of insulin receptors [12]. Vitamin D deficiency has been linked to insulin resistance, and an improvement of insulin action has been observed after vitamin D supplementation [9, 41]. In addition, vitamin D may improve insulin sensitivity by reducing systematic inflammation [42].

Our study has limitations. First, this investigation is cross-sectional, which precludes any causal inference being made on vitamin D status and risk of diabetes. A recent meta-analysis of 11 prospective cohort studies involving a total of 3,612 cases reported a 70 % increased risk of type 2 diabetes in association with vitamin D insufficiency [33], but two randomized controlled trials (RCTs) fail to support vitamin D supplementation prevents diabetes [43, 44]. It is important to note that both trials were conducted in populations where vitamin D deficiency was uncommon. When the benefits from vitamin D supplementation are confined to individuals with vitamin D deficiency, RCTs will not be able to demonstrate additional benefits from supplementation to those with optimal levels [45]. Future longitudinal studies including intervention trials are needed to further elucidate the association between vitamin D and diabetes, in particular in populations where vitamin D deficiency is prevalent. Second, vitamin D status was assessed by a single measurement of serum 25(OH)D and can potentially result in misclassification of subjects’ long-term vitamin D status. However, a recent study that analyzed serum 25(OH)D from the same individual at baseline, year-1 and year-5 observed relatively low within-subject variability and fairly high correlations in serum 25(OH)D measured at the three time points, suggesting serum 25(OH)D is reasonably stable over a long period of time [46]. When misclassification of vitamin D status using a single measurement is present, it is likely to be non-differential, which will result in bias towards the null. The true association would be even stronger than what was observed. Third, several demographic and behavior risks are associated with both vitamin D status and diabetes risk. Although we adjusted a variety of confounders in our analyses, the possibility of residual confounding cannot be ruled out which can either over- or under-estimate the results. Fourth, the survey did not distinguish between type I or type II diabetes although previous studies demonstrated vitamin D can affect both type I diabetes through its immunomodulatory effect [47] and type II diabetes through anti-inflammatory effect [48]. Last, as with any study, our results may be sensitive to bias from non-participation given that not all eligible households participated in the survey. However, sampling weights were adjusted in all analyses to account for the complex survey design including non-participation.

## Conclusions

In a national representative sample of Kuwaiti adults that suffer from a rising incidence of diabetes, we found an alarmingly high prevalence of vitamin D deficiency, and low vitamin D status, is associated with a high prevalence of diabetes. This calls for further investigating the health consequences of low vitamin D status, i.e. serum 25 (OH)D <20 ng/ml in Kuwait and other Middle East countries, and the potential of improving vitamin D status to prevent diabetes at the population level. Given the high prevalence of diabetes and vitamin D deficiency in Kuwaiti adults, improving vitamin D status, even to a small extent, can potentially have a large impact on diabetes prevention in this high-risk population.

## Availability of data and materials

The data supporting the conclusions of this article were based on the National Nutrition Survey of the State of Kuwait (NNSSK). The data are not publically available but researchers who wish to use the data for non-commercial purposes, without breaching participant confidentiality can obtain access to the data by directly contacting the Kuwait Institute for Scientific Research.

## Additional file:

Additional file 1: Table S1. Prevalence of vitamin D inadequacy and deficiency by diabetes status in Kuwaiti adults, NNSSK 2008-2009. **Table S2**. Associations between vitamin D and diabetes by weight status, dietary intake of calcium and vitamin D, and use of calcium supplement in Kuwaiti adults, NNSSK 2008-2009. (DOCX 22 kb)

## Abbreviations

25(OH)D: 25-hydroxyvitamin D
BMI: body mass index
CV: coefficient of variation
HbA1C: hemoglobin A1c
IQR: interquartile range
