# Supplementary material for: Vitamin D deficiency is associated with high prevalence of diabetes in Kuwaiti adults: results from a national survey
Source: BMC Public Health. 2016 Feb 1;16:100. doi: 10.1186/s12889-016-2758-x (PMC4735959; doi:10.1186/s12889-016-2758-x)
Supplement: Additional file 1: Table S1. — Prevalence of vitamin D inadequacy and deficiency by diabetes status in Kuwaiti adults, NNSSK 2008-2009. Table S2. Associations between vitamin D and diabetes by weight status, dietary intake of calcium and vitamin D, and use of calcium supplement in Kuwaiti adults, NNSSK 2008-2009. (DOCX 22 kb) [file 12889_2016_2758_MOESM1_ESM.docx]

**Additional file 1: Table S1.** Prevalence of Vitamin D Inadequacy and Deficiency (serum 25 (OH)D) by Diabetes Status Kuwaiti Adults, NNSSK 2008-2009^1^

| Diabetes Status | Sufficiency  (≥ 20 ng/ml) | Inadequacy  (=12-19.9 ng/ml) | OR (95%CI) | | Deficiency  (<12 ng/ml) | OR (95%CI)^2^ | |
| --- | --- | --- | --- | --- | --- | --- | --- |
|  | N (%) | N (%) | Model I^2^ | Model II^3^ | N (%) | Model I^2^ | Model II^3^ |
|  |  |  |  |  |  |  |  |
| Nondiabetic | 66 (17.0) | 167 (43.0) | 1.0 | 1.0 | 155 (40.0) | 1.0 | 1.0 |
| Prediabetic | 50 (15.4) | 149 (46.0) | 1.9 (1.2-3.0) | 1.6 (1.0-2.7) | 125 (38.4) | 2.2 (1.3-3.6) | 1.9 (1.1-3.2) |
| Diabetic | 48 (19.7) | 131 (53.7) | 2.3 (1.4-3.9) | 1.9 (1.1-3.4) | 65 (26.6) | 2.0 (1.1-3.6) | 1.9 (1.0-3.7) |
|  |  |  | P _trend_=0.002 | P _trend_=0.03 |  | P _trend_=0.01 | P _trend_=0.03 |

1. According to the American Diabetes Association’s criteria for the diagnosis of diabetes,[[2](#_ENREF_2)] a subject was defined as having diabetes if fasting glucose ≥7.0 mmol/L or HbA1c≥6.5%, or if a subject reported a physician’s diagnosis of diabetes or use of medications to control diabetes. A subjects was defined as having prediabetes if fasting glucose = 5.6-6.9 mmol/L or HbA1c = 5.7-6.4%.
2. Model I: Odds ratios (ORs) and 95% confidence intervals (CIs) were adjusted for age.
3. Model II: Odds ratios (ORs) and 95% confidence intervals (CIs) were additionally adjusted for sex, education (less than high school, high school, and college or higher), body mass index (continuous), smoking status (current, former, and nonsmokers), physical activity (active *vs*. sedentary), dietary intake of vitamin D and calcium (high *vs*. low according to median), supplemental intake of vitamin D and calcium (yes *vs*. no), and season of blood draw (summer/spring *vs.* winter/fall).

**Additional file 1 Table S2.** Associations between Vitamin D and Diabetes by Weight Status, Dietary Intake of Calcium and Vitamin D, and Use of Calcium Supplement in Kuwaiti Adults, NNSSK 2008-2009

|  |  | Nondiabetic | Prediabetic  / Diabetic | OR (95%CI) | |
| --- | --- | --- | --- | --- | --- |
|  |  | N (%) | N (%) | Model I^1^ | Model II^2^ |
|  |  |  |  |  |  |
| **Serum 25 (OH)D** | **Overweight/obese** |  |  |  |  |
| Sufficient | No | 28 (7.3) | 16 (2.8) | 1.0 | 1.0 |
| Inadequate / deficient | No | 102 (26.4) | 59 (10.4) | 2.7 (0.9-3.4) | 2.9 (1.3-6.1) |
| Sufficient | Yes | 38 (9.8) | 82 (14.5) | 2.0 (1.0-4.1) | 2.1 (0.9-4.7) |
| Inadequate / deficient | Yes | 218 (56.5) | 409 (72.3) | 3.4 (1.8-6.1) | 3.6 (1.8-7.1) |
|  |  |  |  | P _interaction_=0.46 | P _interaction_=0.21 |
|  |  |  |  |  |  |
| **Serum 25 (OH)D** | **Dietary intake of Vitamin D** | |  |  |  |
| Sufficient | High | 35 (9.3) | 55 (10.0) | 1.0 | 1.0 |
| Inadequate / deficient | High | 148 (39.4) | 225 (40.8) | 1.4 (0.8-2.2) | 1.4 (0.8-2.3) |
| Sufficient | Low | 29 (7.7) | 41 (7.4) | 0.8 (0.4-1.5) | 0.8 (0.4-1.6) |
| Inadequate / deficient | Low | 164 (43.6) | 231 (41.9) | 1.8 (1.1-2.8) | 1.7 (1.0-2.8) |
|  |  |  |  | P _interaction_=0.48 | P _interaction_=0.52 |
|  |  |  |  |  |  |
| **Serum 25 (OH)D** | **Dietary intake of calcium** | |  |  |  |
| Sufficient | High | 37 (9.6) | 67 (11.9) | 1.0 | 1.0 |
| Inadequate / deficient | High | 133 (34.6) | 235 (41.7) | 1.9 (1.1-3.0) | 2.1 (1.2-3.6) |
| Sufficient | Low | 29 (7.5) | 31 (5.5) | 0.7 (0.3-1.5) | 0.9 (0.4-2.0) |
| Inadequate / deficient | Low | 186 (48.3) | 230 (40.9) | 1.7 (1.1-2.8) | 1.7 (1.0-2.9) |
|  |  |  |  | P _interaction_=0.73 | P _interaction_=0.61 |
|  |  |  |  |  |  |
| **Serum 25 (OH)D** | **Use of calcium supplement** | |  |  |  |
| Sufficient | Yes | 13 (3.4) | 15 (2.6) | 1.0 | 1.0 |
| Inadequate / deficient | Yes | 19 (4.9) | 41 (7.2) | 2.4 (1.0-5.9) | 2.8 (1.1-7.4) |
| Sufficient | No | 53 (13.7) | 83 (14.6) | 1.5 (0.7-3.3) | 1.5 (0.7-3.5) |
| Inadequate / deficient | No | 303 (78.1) | 429 (75.7) | 2.8 (1.4-5.5) | 2.8 (1.3-5.8) |
|  |  |  |  | P _interaction_=0.34 | P _interaction_=0.40 |

1. Model I: Odds ratios (ORs) and 95% confidence intervals (CIs) were adjusted for age.
2. Model II: Odds ratios (ORs) and 95% confidence intervals (CIs) were additionally adjusted for sex, education (less than high school, high school, and college or higher), body mass index (continuous), smoking status (current, former, and nonsmokers), physical activity (active *vs*. sedentary), dietary intake of vitamin D and calcium (high *vs*. low according to median), supplemental intake of vitamin D and calcium (yes *vs*. no), and season of blood draw (summer/spring *vs.* winter/fall).
